# Cæsarian Section

**Published:** 1901-10-19

**Authors:** 


### Hospital Clinics and Medical Progress.

### CÆSARIAN SECTION.

The steadily accumulating proofs of the safety of Cæsarian section, when performed under proper conditions, raise many ethical questions in regard to the propriety of other methods of treatment in cases where the birth of a living child by way of the natural passages is impossible. With many men it is a sort of instinct to save the life of the woman even though the child may thereby have to be destroyed. Some, indeed, confronted with the logical difficulty of deciding at what particular moment of its intra-uterine existence the child is to be regarded as a separate individual, with individual rights, have gone to the extreme of denying it all rights until such time as it shall by actual birth have taken on its own separate and independent life. To those who choose to take this view all ethical difficulties vanish. The fœtus whether it be a mere ovum or a fully-grown child is regarded as a mere appendage or outgrowth of the mother, and thus under all circumstances the mother's life must be first considered. This has for long been regarded as the English view of the question. But it must with every man go very much against the grain to destroy life, even though the saving of life be the end in view, and under the influence of religious teaching, and perhaps of certain military leanings which place the possibility of the birth of a boy far above the chance of the loss of a woman, the tendency has been in certain countries to regard craniotomy as a murderous proceeding, and to lean strongly towards Cæsarian section in the treatment of seriously obstructed labour. The dangers of this operation, however, were great and, at any rate in England, it was always regarded as a *dernier ressort*.

With the developments of asepticism and other modern methods, however, the question assumes a new aspect. What was a desperate venture—a proceeding to which operators were driven by necessity, but from which they were often glad to escape even by way of craniotomy—has become an operation which may be compared almost with ovariotomy so far as the peril of the mother is concerned. No longer do we feel that in suggesting Cæsarian section we are asking the mother to stake her life for the sake of her child, for if we are allowed to choose a proper time and to make proper preparations the risk is held by some not to be greater than the statistics show the alternatives to be.

Moreover, there are other considerations which must not be neglected. One of the alternatives, which is very tempting if one is consulted early enough, is the induction of premature labour or even of abortion. By these means the mother gets out of her difficulty and our conscience is spared. But in many cases, even where the birth of a living, viable child is aimed at, the induction must take place at such a date that the chances for the child are but poor; and when it is a matter, not of one pregnancy alone, but of a woman coming every year or so asking for induction of premature labour, a proceeding which will end probably in the production of a non-viable fœtus, or at the best only in the birth of a miserable squab of an "incubator" baby, it becomes a very serious question whether a woman who under such circumstances allows herself to become pregnant should not take the risk of Cæsarian section—nowadays not such a great risk—for the sake of getting at least one well-developed full-time child. So much for the child. For the mother the operation offers the further advantage that, either by the excision of a bit out of each tube or by the removal of the uterus, it may be so arranged as to prevent any further conception taking place; although it is to be noted that some go so far as to question the morality of even this proceeding. In a recent discussion of the whole subject it was held by Dr. Monro Kerr, obstetric physician to the Glasgow Maternity Hospital, that in the present position of operative surgery, and in view of the comparative safety of Cæsarian section when performed under favourable conditions, we are hardly ever justified in destroying a living child by craniotomy or other destructive operation. The only circumstances, he said, under which such operative measures are permissible are where fruitless attempts have been made to extract with forceps or version, where the mother's condition is such as to preclude abdominal section, and where the child is the subject of hydrocephalus or some other extreme malformation, or is on the point of dying. The line of practice thus pointed out is very clear and distinct, and by following it we are faced by no subtle ethical problems. "Some little time ago," he says, "a woman came to me far advanced in her fourth pregnancy. In all her previous labours the child had to be destroyed by craniotomy, and she wished to arrange for the same operation being done for a fourth time. As she refused to submit to Cæsarian section there was nothing for it but to decline to treat her, which I did. No self-respecting man can go on destroying children indefinitely!" If one puts aside all question of the child, and regards the matter, as so many English obstetricians do regard it, as a mere question of the safety of the mother, then one must admit that modern improvements of technique cut both ways, and that while asepticism has worked wonders in lessening the gravity of Cæsarian section, so it, and the use of the cephalotribe in its various modifications, has done much to diminish the dangers attending craniotomy, while the induction of miscarriage or premature labour, which used to be somewhat perilous proceedings, may now be said to be practically devoid of risk. In the discussion above alluded to Dr. Peter Horrocks said that it was not fair to compare modern results of Cæsarian section with mortality statistics of craniotomy, etc., in pre-aseptic days. Practically, he said, induction of miscarriage and labour, and craniotomy, were all so safe nowadays as to have no mortality. Thus the problem comes back to the old point of a comparison in each individual case of the risks of the various proceedings which are open to us. When modern improvements are pointed to as favouring one course we must not forget that they also, perhaps in even greater degree, favour the other, and we shall most of us probably agree with what Dr. Horrocks said, namely, that we are hardly yet in a position to decline to bring on a miscarriage or to induce a premature labour, or to perform craniotomy upon a patient who refuses to submit to Cæsarian section. The time may come when one may have to take up such a position, but that time has hardly yet arrived.

